# Magnetic resonance imaging findings in primary lymphoma of the liver: a case report

**DOI:** 10.1186/1752-1947-6-282

**Published:** 2012-09-05

**Authors:** Fatmir Bilaj, Leart Berdica, Arben Dhima, Gjergji Vreto

**Affiliations:** 1 Department of Radiology, “Medicare” Diagnostic Center, University Hospital Center “Mother Teresa”, Tirana, Albania; 2 Department of Anatomo-pathology, University Hospital Center “Mother Teresa”, Tirana, Albania

## Abstract

**Introduction:**

Primary lymphoma of the liver is an extremely rare finding, with the few such cases reported in the literature to date describing indeterminate imaging findings, being focused more on computed tomography. To the best of our knowledge, there is no prior report describing magnetic resonance imaging scan findings with such a lesion. In the case reported here, magnetic resonance imaging gave us the opportunity to ascertain the correct diagnosis, confirmed by histopathology, thus avoiding unnecessary surgery or other treatments. Although this condition is rare, knowledge of magnetic resonance imaging findings will be invaluable for radiologists and other medical subspecialties that may face such cases in the future in helping to provide adequate management for affected patients.

**Case presentation:**

A focal lesion was incidentally detected by ultrasound in a 75-year-old asymptomatic Albanian man being treated for benign hypertrophy of prostate. Chest and abdomen computed tomography scans did not reveal any abnormal findings besides a solid focal lesion on the right lobe of the liver and a mild homogenous enlargement of the prostate gland. Subsequently, magnetic resonance imaging of the upper abdomen was performed for better characterization of this lesion. Our patient was free of symptoms and his laboratory test results were normal.

**Conclusions:**

The magnetic resonance imaging scan results showed some distinctive features that helped us to make the correct diagnosis, and were thus very important in helping us provide the correct treatment for our patient.

## Introduction

Primary lymphoma of the liver is extremely rare. There are few prior cases describing the imaging findings of such lesions. Most of these articles to date are based on computed tomography (CT) findings, and to the best of our knowledge there are no articles describing magnetic resonance imaging (MRI) findings in any detail [1-3]. In this case we present imaging, clinical and laboratory findings, and a review of the literature. Our patient was free of symptoms and no further treatment was recommended. The lesion was unchanged based on the follow-up MRI scans performed every six months for the next two years.

## Case presentation

We report the MRI findings of an incidental focal liver lesion, which was detected by ultrasound (US) in a 75-year-old Albanian man treated for benign hypertrophy of the prostate. His physical examination and laboratory test results were unremarkable.

Multi-detector chest and abdominal computed tomography (MDCT), with intravenous administration of 100mL iodinated contrast material was performed using a Emotion 6 MDCT device (Siemens Medical Systems, Erlangen, Germany). MDCT scans showed a focal liver lesion in the right lobe, and a mild enlargement of the prostate gland. No other abnormalities were identified in the rest of his chest and abdomen. Subsequently, an MRI scan of the upper abdomen was performed for better characterization of this lesion.

An MRI scan was performed using a 1.5T magnetic resonance unit (Avanto; Siemens Medical Systems, Erlangen, Germany), using a set protocol including unenhanced sequences and enhanced sequences, obtained in early phase at 25 seconds after administration of intravenous contrast gadolinium (Magnevist, Bayer HealthCare) at 2mL/second, 45 and 90 seconds (late phase).

Two-dimensional gradient echo in axial plane was performed using these parameters: TR/TE 140 to 200/2.2 to 2.4ms, flip angle 80, slice thickness 6mm, image matrix 128 × 256 and acquisition time 20 seconds.

A three-dimensional gradient echo sequence (VIBE) with a fat saturation was performed before and after administration of intravenous contrast using the following parameters: 5.58/2.38ms, flip angle 10, matrix 320 × 256, slice thickness 3.3mm and acquisition time 20 seconds.

A dual T1-weighted spoiled gradient echo (SGE) sequences in phase/out-of-phase was obtained in the axial plane using the following parameters: 113/4.92/2.22ms, flip angle 70, slice thickness 8mm and acquisition time 22 seconds for each sequence.

T2-weighted half-Fourier acquisition single-shot turbo spin echo (HASTE) with and without fat saturation was obtained using the following parameters: 1000/94ms, flip angle 15, slice thickness 6mm, matrix 320 × 246 and acquisition time 22 seconds.

## Discussion

To make a diagnosis of primary lymphoma of the liver by imaging is a challenging task because of the rarity of such lesions and indeterminate imaging findings of the same published to date [1]. The differential diagnosis includes a broad range of solitary solid lesions.

The primary concern is hepatocellular carcinoma (HCC). HCC develops in 80% of cases in the cirrhotic liver, mainly in patients who test positive for hepatitis B or C, in alcoholic cirrhosis or in a combined form; it is less likely in other cases with primary biliary cirrhosis, hemochromatosis, or cryptogenic cirrhosis.

Our patient was free of symptoms: his laboratory test results did not suggest liver damage.

His α-fetoprotein (AFP) level was within the normal range; our patient was not alcoholic, and he tested negative for hepatitis B and C.

His MRI scan results did not show morphologic changes favoring cirrhosis. Even in the absence of risk factors, HCC of this size is usually a hyper-vascular tumor that shows marked homogeneous enhancement in the arterial phase with late washout that is presented with capsular enhancement [2]. The appearance of the lesion on MRI scan was different, so HCC was excluded.

Liver metastasis was the next concern for our patient. However, our patient did not have any known history of a primary tumor. Additionally, all test results for tumor markers were negative.

The MRI appearance of metastases is variable in T1-weighted and T2-weighted images, but metastases usually possess a ring enhancement in the delayed phase after administration of intravenous contrast [3]. Due to the lack of this type of enhancement with no primary tumor history and with all tumor markers testing negative, this made metastasis unlikely as well.

Secondary liver involvement by lymphoma is common. Such lesions are usually multiple and resemble metastases. It is often associated with spleen involvement and enlarged lymph nodes along the lymphatic chain of the abdomen and mesentery. The appearance on CT and MRI is variable, and does not reveal any signs typical for liver infiltration by lymphoma. Knowing the history of lymphoma is pivotal to making the right characterization [4].

Our patient’s chest and abdominal CT scans did not show any enlarged lymph nodes, and our patient did not have any history of prior known lymphoma. The liver lesion was solitary and did not look like a metastasis. In this setting secondary lymphoma of the liver was entirely excluded.

A mesenchymal lesion, representing as a focal mass, should be the other major differential diagnosis, especially inflammatory myofibroblastic tumor and unusual mesenchymal tumor.

The MRI appearance of inflammatory myofibroblastic tumor is equivocal and is often accompanied by soft tissue infiltrating the periportal space. Characterization of these lesions even with MRI is difficult. When they are suspected, a biopsy is needed to make a definitive diagnosis [5].

Mesenchymal tumors of the liver are very rare, and their appearance is similar to malignant epithelial tumor. Some of them possess distinctive features that may help to suggest the correct diagnosis, but in most cases, the definitive diagnosis is made through histopathological examination [6].

**Figure 1:**
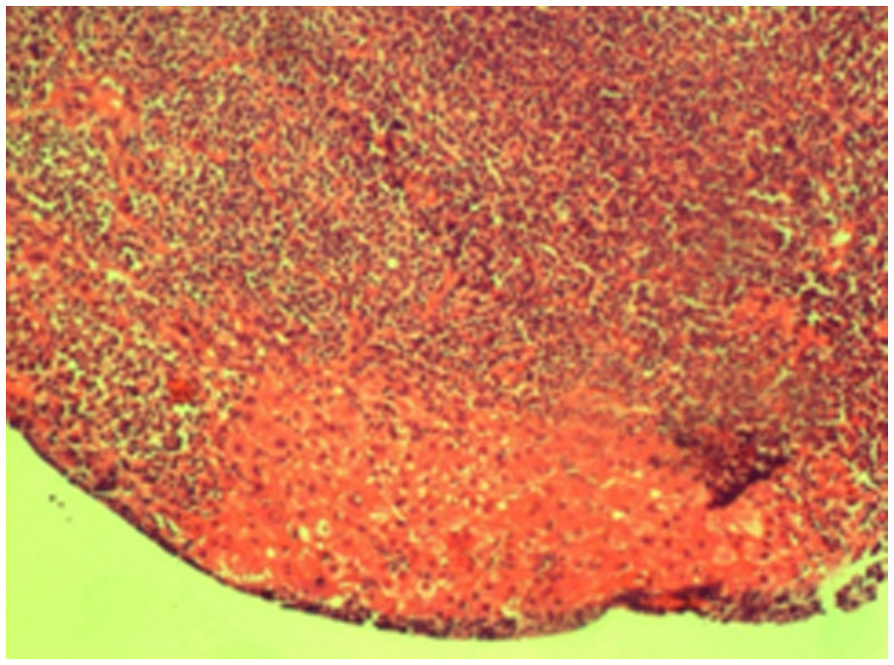
**Photograph of the pathology specimen.** The figure shows a histopathology section of the liver obtained through ultrasound (US)-guided biopsy, showing the lymphoid tissue infiltrating the liver parenchyma. The lymphoid tissue is composed of small lymphocytes with oval and irregular nuclei and clear cytoplasm (hematoxylin and eosin stain, ×40).

Our patient underwent ultrasound-guided biopsy, and pathology revealed a low-grade B cell lymphoma. The pathology specimen showed marginal infiltration by B cells, which on testing were immunohistochemically positive for CD20 and negative for CD10, CD3, CD43 and B cell lymphoma 2 proteins (Bcl-2) (Figure1).

The rest of our patient’s chest and abdominal CT results did not show any nodal involvement, or any other organ infiltration. His laboratory test results were within normal ranges.

Primary lymphoma of the liver is defined as a disease where the involvement is confined to the liver or portal hepatic lymph nodes, without involvement of the spleen, other lymph nodes or bone marrow. It accounts for less than 1% of extranodal lymphomas. The causes are not well known, and there is no wide consensus on treatment [7]. It usually affects patients in the seventh to eighth decade of life and is more common in men, with a male to female ratio of 4:1.

It is located more often in the right lobe that in the left lobe. There is an increasing incidence of cases in patients who are immunocompromised. Even the pathological features are very variable and additional clinical findings are important in making the right diagnosis [8]. Patients usually complain of right flank pain, anorexia, and diaphoresis or weight loss. Our patient was free of symptoms and no further treatment was recommended.

The lesion was followed-up for a two-year period, with MRI scans performed every six months for up to two years; in that time the lesion remained unchanged.

There are a few radiological reports describing the imaging findings of primary lymphoma of the liver. CT scans usually show a large hypodense lesion, which shows mild enhancement after administration of intravenous contrast. The lesions can be solitary or multiple and only in one previous case showed a marked enhancement. Some lesions contain an area of necrosis or calcification [9].

MRI, because of its superior soft tissue discrimination, gives more information about the internal structure of these lesions and mode of enhancement. On MRI, these lesions are usually low signal in T1, moderately high signal in T2, demonstrating mild enhancement with target appearance in the one hour late phase, composed of a central enhancement portion surrounded by a hypointense rim. This is a unique feature, different from malignant tumors arising from hepatocytes [10].

**Figure 2:**
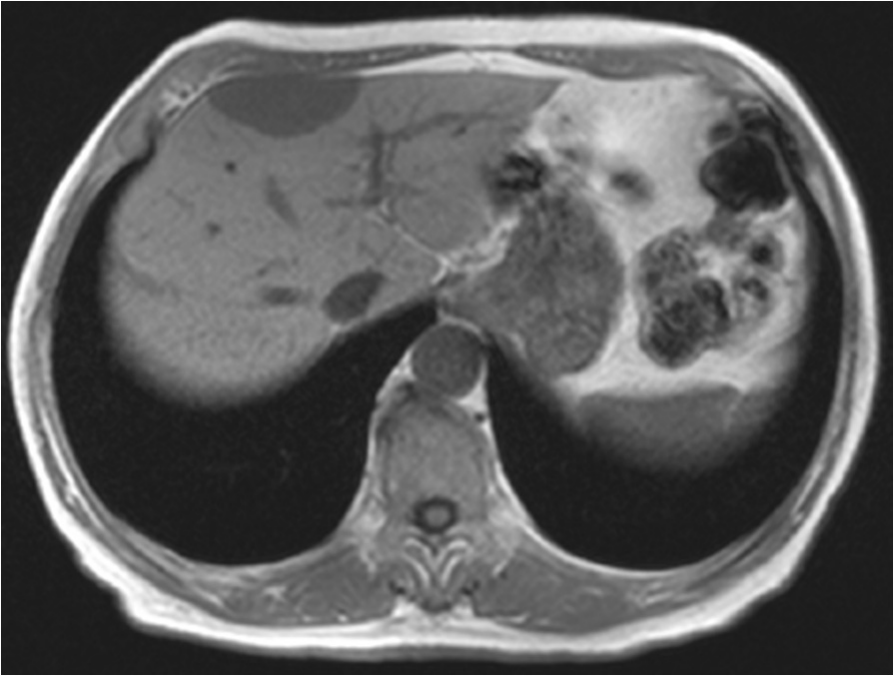
Axial in-phase gradient echo (GRE) T1-weighted magnetic resonance imaging (MRI) scan, obtained as a part of dual GRE (chemical shift) sequences, showing a focal mass in the right lobe to be hypointense when compared to liver parenchyma, but iso-intense compared to spleen parenchyma.

**Figure 3:**
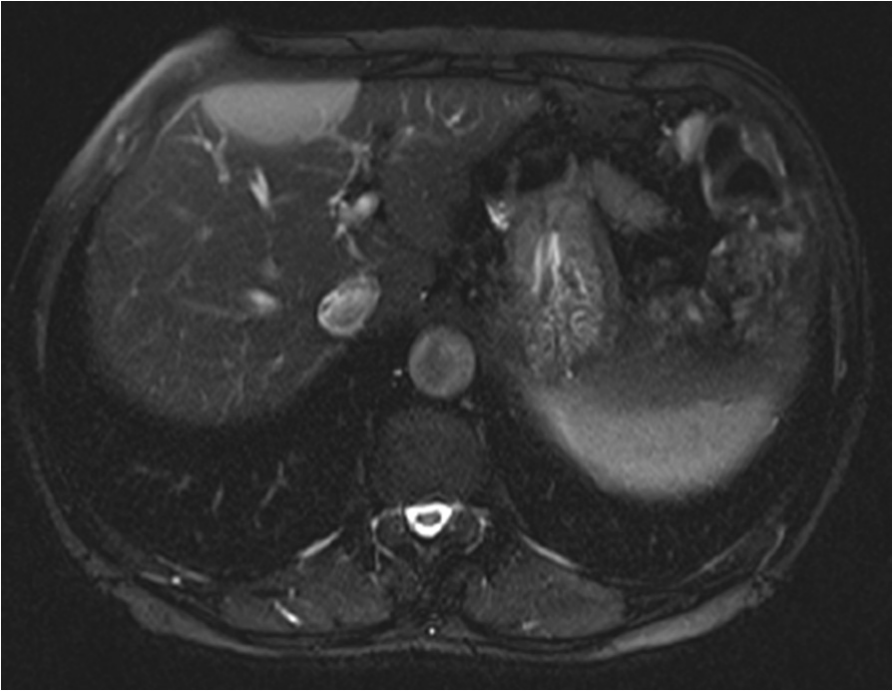
Axial T2-weighted with fat suppression magnetic resonance imaging (MRI) scan showing a mass that demonstrates moderate homogenous hyperintensity, but that is iso-intense when compared with spleen parenchyma.

**Figure 4:**
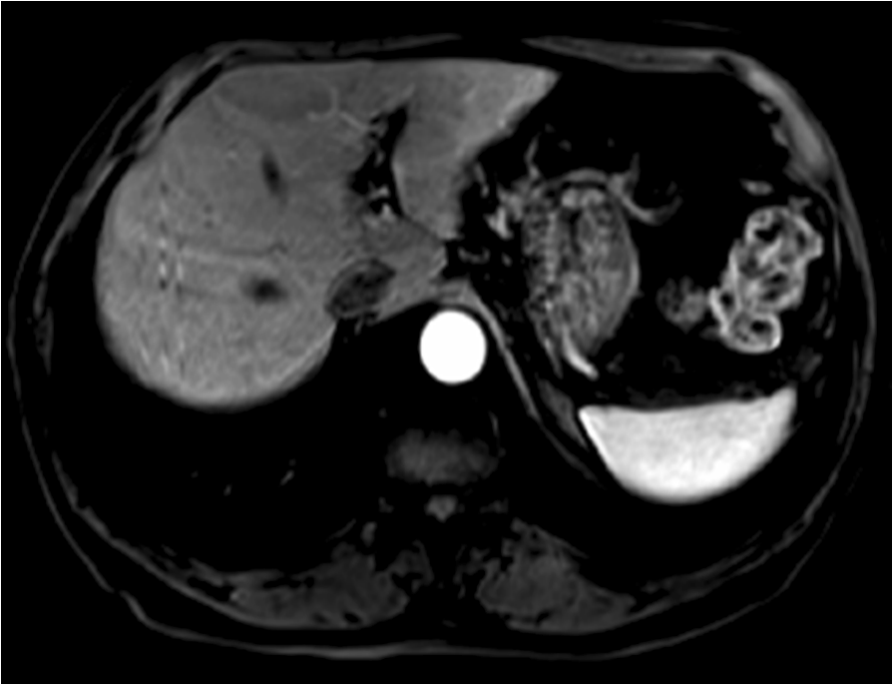
Axial three-dimensional arterial phase gradient echo (GRE) T1-weighted images demonstrating mild homogeneous enhancement of the mass.

**Figure 5:**
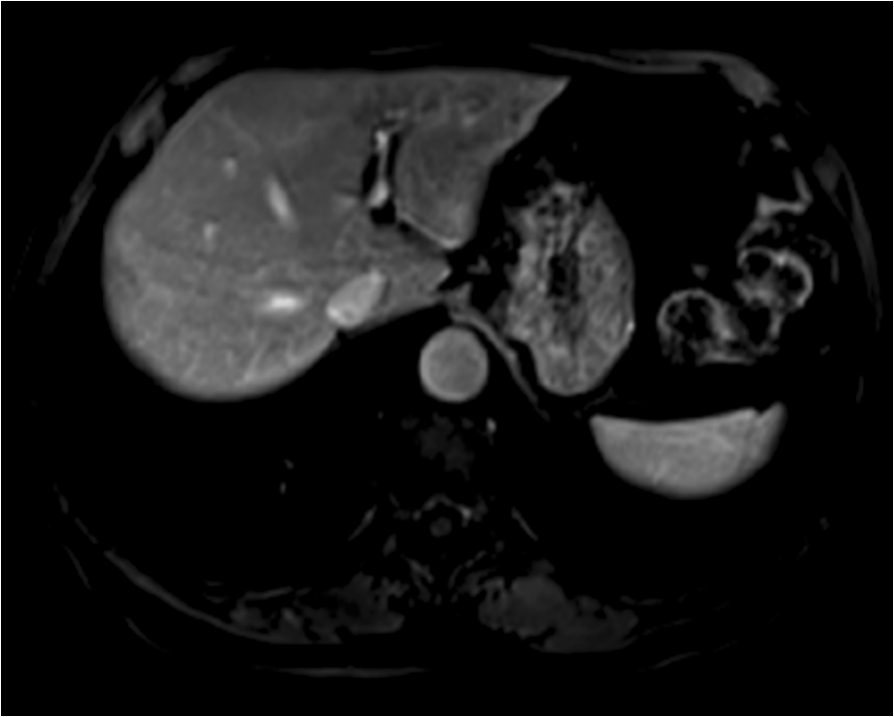
Axial three-dimensional late phase gradient echo (GRE) T1-weighted images, obtained 90 seconds after administration of intravenous contrast, demonstrating perilesional enhancement of the mass.

In our patient’s case, we observed some distinctive features that helped us to make the correct diagnosis. The lesion was iso-intense with spleen parenchyma on both T1-weighted and T2-weighted images in spite of signal intensity compared with liver parenchyma (Figure2, 3). It showed mild homogenous enhancement in the arterial phase and some mild perilesional enhancement in the delayed phase obtained 90 seconds after administration of intravenous contrast (Figure4, 5).

## Conclusions

This case illustrates the MRI findings of primary lymphoma of the liver. MRI provides some distinctive features, such as iso-intensity with spleen parenchyma in both T1-weighted and T2-weighted images and mild perilesional enhancement in delayed images. Although these cases are very rare, knowledge of these imaging findings will be helpful for radiologists to suggest the correct diagnosis and other medical subspecialties to provide adequate treatment.

## Consent

Written informed consent was obtained from the patient for publication of this case report and accompanying images. A copy of the written consent is available for review by the Editor-in-Chief of this journal.

## Competing interests

The authors declare that they have no competing interests.

## Authors’ contributions

FB analyzed, interpreted the MRI scans and was the major contributor to the writing of the manuscript. LB performed the histological examination of the material taken from our patient’s liver through ultrasound-guided biopsy. ADH performed research in PubMed, and helping in writing the paper. GJV reviewed the manuscript and prepared the final draft. All authors read and approved the final manuscript.
